# Magnetic resonance imaging findings of cellular angiofibroma of the tunica vaginalis of the testis: a case report

**DOI:** 10.1186/s13256-016-0861-3

**Published:** 2016-03-31

**Authors:** Alexandra A. Ntorkou, Athina C. Tsili, Dimitrios Giannakis, Anna Batistatou, Sotirios Stavrou, Nikolaos Sofikitis, Maria I. Argyropoulou

**Affiliations:** 1 Department of Clinical Radiology, Medical School, University of Ioannina, 45110 Ioannina, Greece; 2 Department of Urology, Medical School, University of Ioannina, 45110 Ioannina, Greece; 3 Department of Pathology, Medical School, University of Ioannina, 45110 Ioannina, Greece

**Keywords:** Cellular angiofibroma, Diffusion-weighted MRI, Magnetic resonance, Magnetization transfer contrast imaging, Tunica vaginalis

## Abstract

**Background:**

Cellular angiofibroma represents a rare mesenchymal tumor typically involving the inguinoscrotal area in middle-aged men. Although the origin of this benign tumor is unknown, it is histologically classified as an angiomyxoid tumor. Cellular angiofibroma is characterized by a diversity of pathological and imaging features. An accurate preoperative diagnosis is challenging. Magnetic resonance imaging examination of the scrotum has been reported as a valuable adjunct modality in the investigation of scrotal pathology. The technique by providing both structural and functional information is useful in the differentiation between extratesticular and intratesticular diseases and in the preoperative characterization of the histologic nature of various scrotal lesions. There are few reports in the English literature addressing the magnetic resonance imaging findings of cellular angiofibroma of the scrotum and no reports on functional magnetic resonance imaging data. Here we present the first case of a cellular angiofibroma arising from the tunica vaginalis of the testis and we discuss the value of a multiparametric magnetic resonance protocol, including diffusion-weighted imaging, magnetization transfer imaging and dynamic contrast-enhanced magnetic resonance imaging in the preoperative diagnosis of this rare neoplasm.

**Case presentation:**

A 47-year Greek man presented with a painless left scrotal swelling, which had gradually enlarged during the last 6 months. Magnetic resonance imaging of his scrotum displayed a left paratesticular mass, in close proximity to the tunica vaginalis, with heterogeneous high signal intensity on T2-weighted images and no areas of restricted diffusion. The tumor was hypointense on magnetization transfer images, suggestive for the presence of macromolecules. On dynamic contrast-enhanced magnetic resonance imaging the mass showed intense heterogeneous enhancement with a type II curve. Magnetic resonance imaging findings were strongly suggestive of a benign paratesticular tumor, which was confirmed on pathology following lesion excision.

**Conclusions:**

Magnetic resonance imaging of the scrotum by combining conventional and functional magnetic resonance data provides useful diagnostic information in the preoperative characterization of scrotal masses. A possible diagnosis of a benign paratesticular tumor based on magnetic resonance imaging features may improve patient care and decrease the number of unnecessary radical surgical explorations.

## Background

Cellular angiofibroma (CA) or angiomyofibroblastoma (AMF)-like tumor is a rare benign mesenchymal tumor, typically involving the genital area of both genders. CA was first described by Nucci *et al.* in 1997 and later by Laskin *et al.* in 1998 as a rare tumor arising in the paratesticular space in men and in the vulva in women [1, 2]. Although the origin of this tumor is not clear, it has been proposed that it derives from perivascular stem cells with potential to differentiate into fatty and myofibroblastic tissue [1–8]. Only a few cases of CA of the scrotum have been reported to date [3–8].

Magnetic resonance imaging (MRI) of the scrotum has been proposed as a valuable alternative imaging technique for the evaluation of scrotal pathology [9–15]. Recently, functional MRI, including diffusion-weighted imaging (DWI), magnetization transfer imaging (MTI) and dynamic contrast-enhanced (DCE) MRI have added important additional diagnostic information in the interpretation of scrotal diseases [9–14]. Here we present the first case of a CA of the tunica vaginalis of the testis, evaluated with a multimodal magnetic resonance (MR) protocol, including DWI, MTI and DCE MRI.

## Case presentation

A 47-year-old Greek man presented to our hospital with a painless left scrotal swelling, which he had for 2 years and which had gradually enlarged during the last 6 months. No prior history of trauma or genitourinary tract infection was reported. A physical examination revealed a painless, firm scrotal mass, associated with hydrocele. No abnormal skin changes were observed. Laboratory tests, including serum tumor markers were unremarkable.

Sonographic examination of his scrotum showed the presence of a large left extratesticular mass, of heterogeneous echotexture. Significant hydrocele was also detected ipsilaterally. Color Doppler assessment revealed rich lesion vascularity.

An MRI examination was performed on a 1.5-T magnet, with the use of a circular surface coil. The MR protocol included axial spin-echo T1-weighted sequences and fast spin-echo T2-weighted sequences in three orthogonal planes. Transverse diffusion-weighted (DW) images were obtained using a single shot, multi-slice, spin-echo planar sequence with *b*-values of 0 and 900 second/mm^−2^. MTI was performed in the axial plane using a three-dimensional gradient-echo sequence, both with and without an on-resonance binomial prepulse to saturate the broad resonance of immobile macromolecular protons. The magnetization transfer ratio (MTR) was calculated as follows: SIo–SIm/SIo × 100, where SIm and SIo refer to signal intensities with and without the saturation pulse, respectively. Coronal DCE subtraction MR images were also obtained after an intravenous injection of gadopentetate dimeglumine, with the use of a three-dimensional fast field-echo sequence. The patterns of contrast enhancement of both his normal testis and the extratesticular lesion were evaluated and time–signal intensity (TSI) curves were created.

**Fig. 1:**
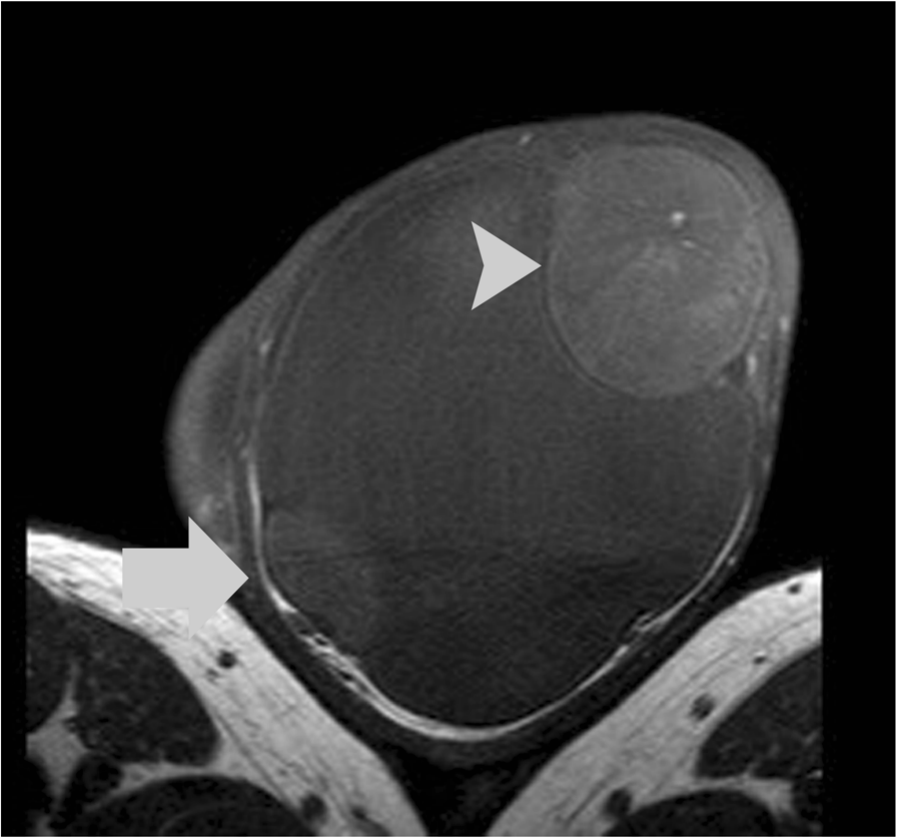
Transverse T1-weighted image shows a well-demarcated left paratesticular mass, lying adjacent to the scrotal wall. The lesion (*arrowhead*) demonstrated mainly similar signal intensity, when compared to the ipsilateral displaced testis (*arrow*). Significant left hydrocele (*long arrow*) was also observed

**Fig. 2:**
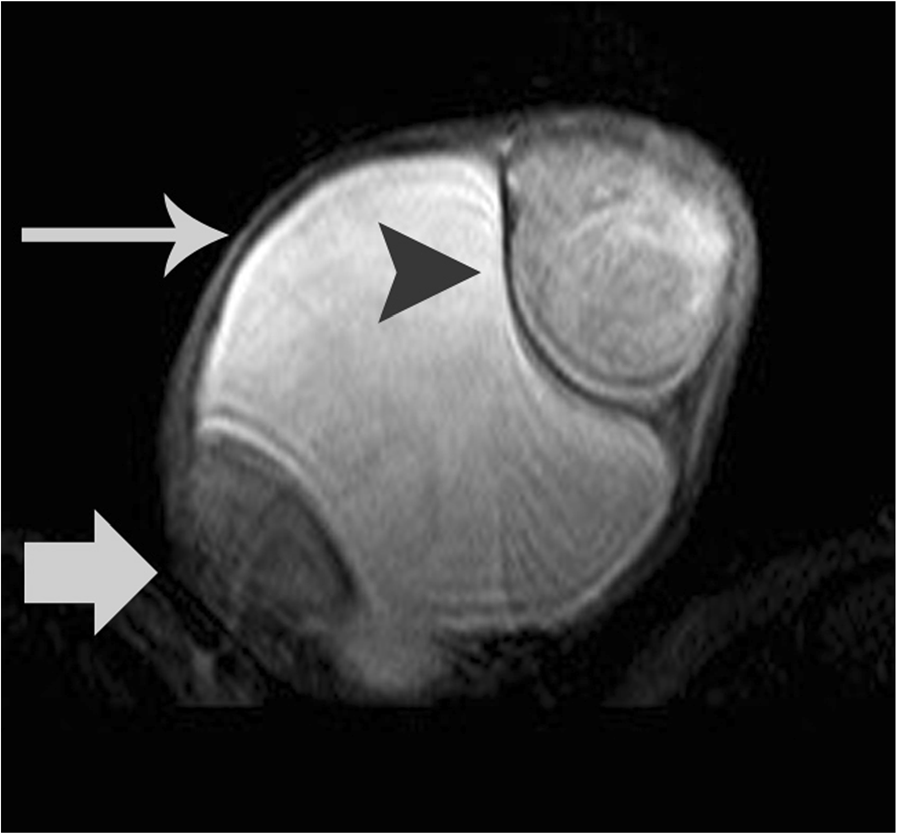
Transverse T2-weighted image demonstrates left paratesticular tumor heterogeneity. The mass (*arrowhead*) was slightly hyperintense when compared to the ipsilateral testicular parenchyma (*arrow*). Left hydrocele (*long arrow*)

**Fig. 3:**
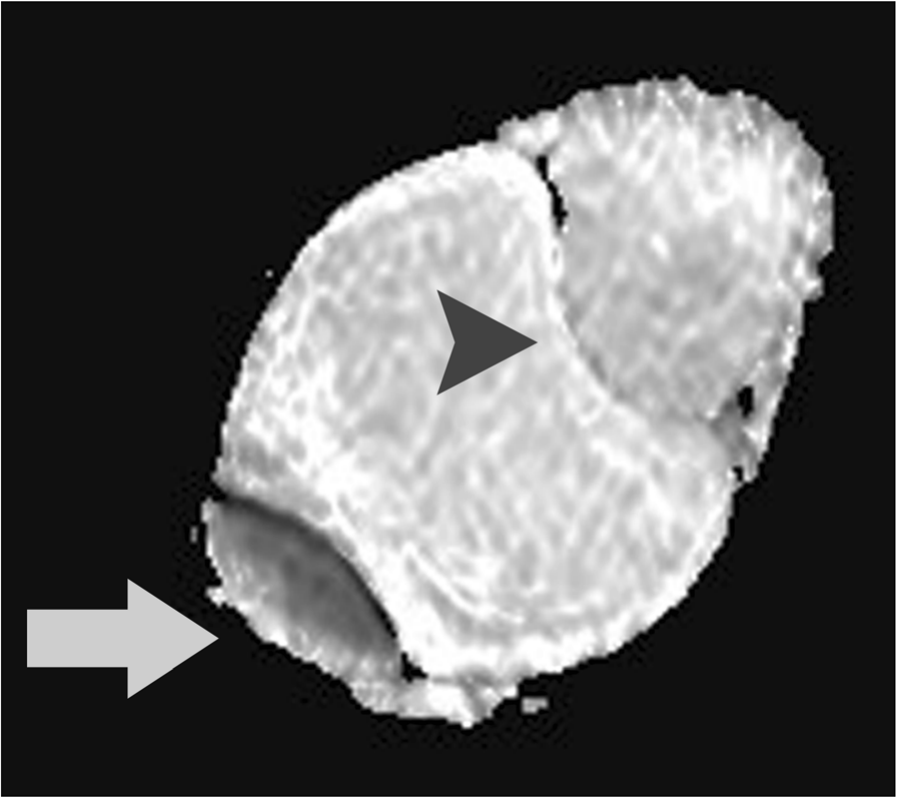
Apparent diffusion coefficient map in axial plane (*b* = 900 mm^2^/second). The lesion appears hyperintense, a finding suggestive for the absence of restricted diffusion. The apparent diffusion coefficient value of the mass (*arrowhead*) was 2.34×10^−3^ mm^2^/second. The apparent diffusion coefficient values of left (*arrow*) and right testes (not shown on image) were 0.99×10^−3^ mm^2^/second and 1.04×10^−3^ mm^2^/second, respectively. The presence of densely packed seminiferous tubules lined by a compact fibroelastic connective tissue sheath and separated by cellular interstitial stroma explains the restricted diffusion of the normal testis. Large left hydrocele

**Fig. 4:**
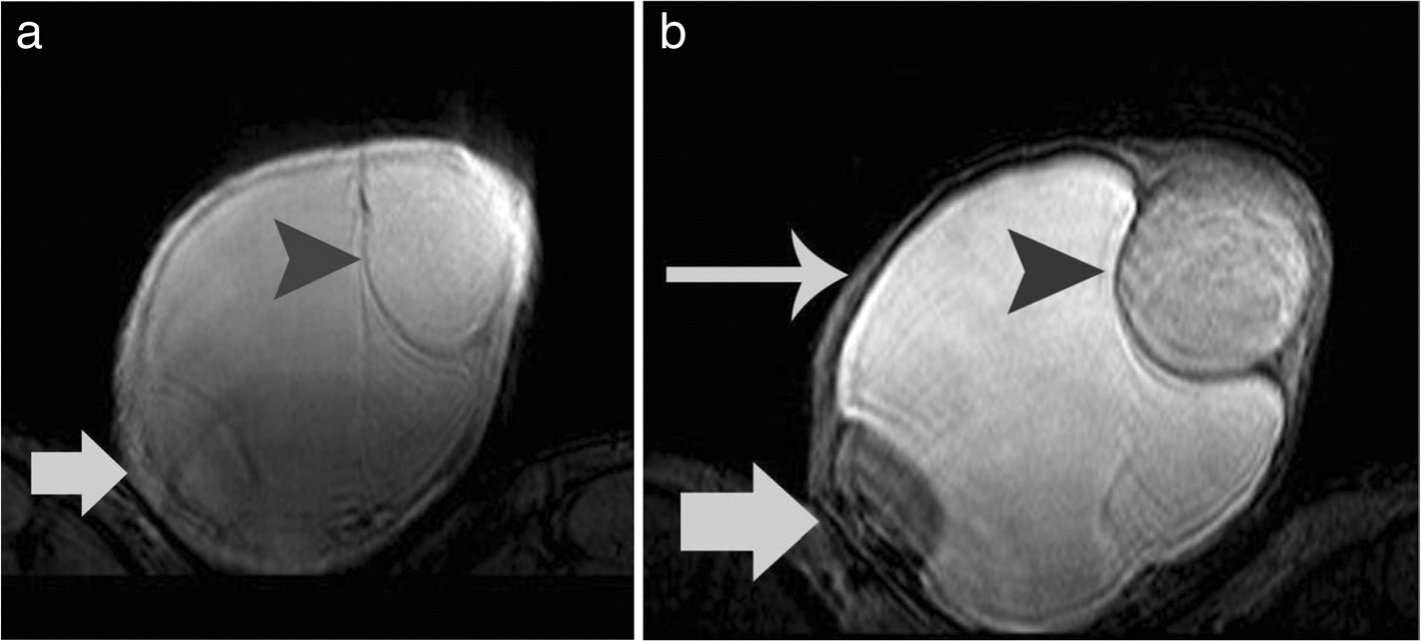
Axial three-dimensional gradient-echo magnetic resonance image before (**a**) and after (**b**) the application of the magnetization transfer prepulse. The magnetization transfer ratio (in percent) of the left paratesticular mass (*arrowhead*) was 44.6, similar to that of the contralateral normal testis (46 %, not shown on images). The left testis (*arrow*) was displaced and compressed and the measurement of the magnetization transfer ratio was impossible due to artifacts. Left hydrocele (*long arrow*)

**Fig. 5:**
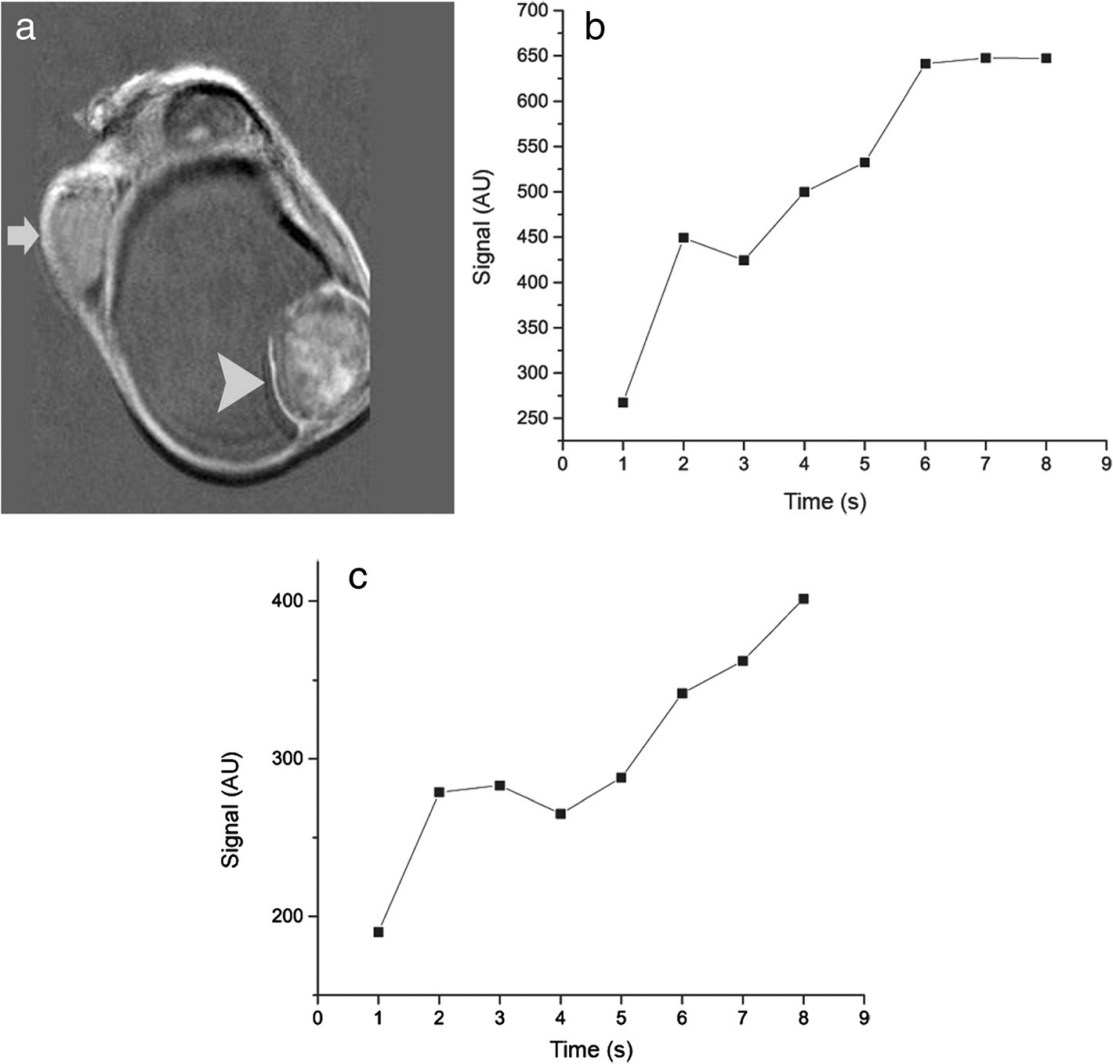
**a** Coronal dynamic contrast-enhanced subtracted image at early phase (180 seconds) and the corresponding time–signal intensity curves of the **b** left paratesticular mass and the **c** ipsilateral testis. The tumor (*arrowhead*, **a**) enhanced strongly and heterogeneously, showing an initial upstroke followed by a plateau in the late contrast-enhanced phase (type II curve, **b**). Dynamic contrast-enhanced magnetic resonance findings were strongly suggestive of benignity. Left testis (not shown on image) showed a linear increase of contrast enhancement throughout the examination (type I curve, **c**). Normal contralateral testis (*arrow*, **a**). *AU* arbitrary units

His spermatic cords, epididymis, and right testis were normal. A well-demarcated left paratesticular mass (Figs. 1, 2, 3, 4, and 5) in close proximity to his tunica vaginalis, displacing his ipsilateral testis was detected, measuring 5.5×4.8×4.3 cm. The tumor was inhomogeneous, mainly with signal intensity similar and slightly higher than that of his normal testis on T1 (Fig. 1) and T2-weighted (Fig. 2) images, respectively. No areas of restricted diffusion were noted on DW images (Fig. 3). Magnetization transfer (MT) images showed low signal intensity for both the paratesticular tumor and the normal testis, suggestive for the presence of macromolecules (Fig. 4). On DCE sequences, the mass showed strong heterogeneous enhancement (Fig. 5a) with a late peak, followed by a plateau in the late contrast-enhanced period (type II curve, Fig. 5b). His left testis enhanced moderately and homogeneously with a linear increase of signal intensity during the entire dynamic period (type I curve, Fig. 5c). MRI findings were strongly suggestive of a benign paratesticular tumor.

**Fig. 6:**
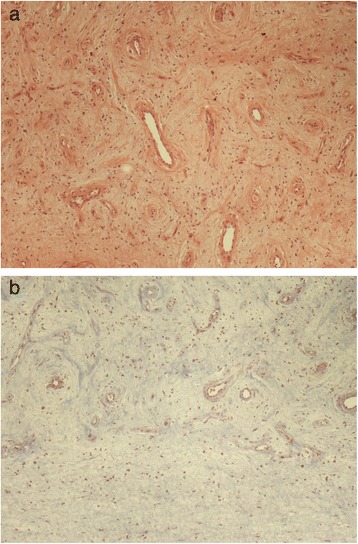
**a** Histopathologic evaluation revealed a highly vascularized tumor, composed of bland spindle-shaped cells (hematoxylin and eosin ×100). **b** The stroma was mostly myxoid (periodic acid–Schiff – Alcian blue ×100)

At left inguinal exploration a hard mass was seen in his paratesticular region, separated from his testis and enucleation of the lesion was followed. Histopathology revealed a neoplasm confined to the parietal lamina of the tunica vaginalis, of low-to-moderate cellularity with abundant myxoid stroma (Fig. 6). Characteristically, bland spindle cells surrounding a well-developed vasculature were noted. The mitoses were extremely rare. The stroma was collagenous and myxoid and contained numerous small to medium-sized blood vessels, with areas of perivascular hyaline fibrosis. There was also lymphocytic infiltration with formation of lymphoid follicles. No areas of hemorrhage or necrosis were detected. On immunohistochemical examination, the neoplastic cells were positive for vimentin, CD31, CD34, estrogen and progesterone receptors, and focally for desmin and smooth muscle actin. With immunostaining for Ki67 it was revealed that less than 2 % of neoplastic cells were in cell cycle. Based on the above, the diagnosis of CA of the tunica vaginalis was made.

The patient had an uneventful recovery and remains well, with no evidence of local recurrence, 10 months after surgery.

## Discussion

CA or AMF-like tumor is a rare benign mesenchymal tumor, usually seen during the fifth to eighth decades of life [1–8]. The majority of these neoplasms arise in the subcutaneous tissue of the genital area, showing no sex predilection. In males, the most common site of origin is the inguinal area, followed by the scrotum and perineum [2–8]. CA arising from the tunica vaginalis of the testis has never been reported in the English-language literature.

Most patients are asymptomatic, presenting with a slow-growing, painless scrotal mass, as it was in this case. Pathological features typically include bland spindle-shaped cells, a myxoid stroma rich in collagen fibers and numerous small to medium-sized blood vessels [1–8]. The recommended treatment is complete surgical removal, with long-term follow-up, as local recurrence has been reported [5–8].

An expanded role for MRI in the evaluation of scrotal pathology has recently been established [9–15]. Conventional MR sequences have been proved reliable in differentiating between extratesticular and intratesticular mass lesions [9, 14, 15]. In many cases, MRI may also provide a preoperative characterization of the histologic nature of various scrotal lesions, including paratesticular masses in terms of morphologic information and tissue signal intensity [9, 15]. The majority of paratesticular tumors are benign and therefore radical orchiectomy may be obviated.

Imaging features of CAs are correlated to their histology, depending on the amounts of spindle cells, myxoid and collagenous stroma and fat within the tumor. A few references have described the MRI findings of CA [5–7]. All lesions were sharply defined, demonstrating invariably heterogeneous high signal intensity on T2-weighted images and signal intensity similar to that of the muscles on T1-weighted images. After gadolinium administration, intense heterogeneous enhancement has been reported [5–7]. The above findings were met in our patient. The presence of abundant myxoid stroma and numerous tumoral vessels probably account for lesion hyperintensity on T2-weighted images and the patterns of contrast enhancement, respectively. However, conventional MRI findings are usually non-specific. Differential diagnosis should include aggressive angiomyxoma, AMF, solitary fibrous tumor, spindle-cell lipoma, well-differentiated liposarcoma and myxoid liposarcoma [1–8].

The presence of intratumoral fat has been reported in 24 to 56 % of CAs [2–7]. Miyajima *et al.* concluded that in the presence of a well-circumscribed hypervascular mass containing fat in the inguinal region of a male the diagnosis of CA should be considered [5]. No areas of macroscopic fat were observed within the neoplasm in our patient.

In our case, a multiparametric MR protocol was used to evaluate the paratesticular mass, including DWI, MTI and DCE MRI. DWI is an evolving technique that can be used to improve tissue characterization when interpreted in combination with conventional MRI findings [9–11]. Lesion detection and characterization on DWI is primarily dependent on the extent of tissue cellularity, and increased cellularity, mostly seen in malignancies, is associated with restricted diffusion and reduced apparent diffusion coefficient (ADC) [9–11]. By combining high *b*-value DWI with conventional MRI findings a high accuracy has been reported in differentiating between normal, benign and malignant scrotal contents [9–11]. Maruyama *et al.* reported a case of a 72-year old man with AMF-like tumor of the scrotum, without areas of restricted diffusion on high *b-*value DW images [6]. In our patient, his paratesticular tumor had high ADC, a finding highly suggestive of the benign nature of the lesion.

MTI provides a different image contrast compared to the conventional MR sequences, which depends mainly on the concentration of macromolecules [12]. Protons in tissues are divided into free water protons and restricted protons, which are bound to proteins and macromolecules. The MT phenomenon is determined by the restricted macromolecular protons and is quantified by the MTR [13]. There is very limited experience in using MTI in the evaluation of scrotal pathology [12]. In the normal testis high MTR values related to macromolecular structures implicated with the secretory activity have been previously reported [12]. In the present case, CA appeared hypointense on MT images, with an MTR similar to that of normal testicular parenchyma, and this could probably be explained by the high collagen content seen on histology. Collagen has been reported as an important determinant of relaxation times in MTI, with large molecular size and extensive intramolecular and intermolecular cross-linking being the characteristics of collagen responsible for the MTI effect [12].

DCE MRI provides information regarding the characteristics of microvasculature of testicular carcinomas, assessing tumor angiogenesis [13, 14]. DCE subtracted MRI has been proved useful in differentiating testicular neoplasms and benign intratesticular lesions [9, 13, 14]. In a previous study, we classified the progression of enhancement of testicular lesions according to the shape of the TSI curves into three types: Type I curve, with a gradual increase of contrast enhancement during the entire dynamic period, found to represent patterns of enhancement of normal testis; type II curve, with an initial upstroke, after which the signal intensity either plateaus or gradually increases in the late contrast-enhanced period, suggestive of a benign diagnosis; and type III curve, with an initial upstroke, followed by gradual washout of the contrast medium, indicating the diagnosis of malignancy [13]. In our patient, CA showed intense heterogeneous enhancement, followed by a plateau at the delayed phase (type II curve), closely correlating to the diagnosis of benignity.

## Conclusions

MRI by combining conventional and functional data may provide valuable information in the pre-surgical work-up, helping in the characterization of the benign nature of paratesticular tumors. A possible diagnosis of benignity based on MRI features may obviate unnecessary radical orchiectomies. A multiparametric MR protocol, including DWI, MTI and DCE MRI may provide important additional diagnostic information in the interpretation of scrotal pathology.

## Consent

Written informed consent was obtained from the patient for publication of this case report and accompanying images. A copy of the written consent is available for review by the Editor-in-Chief of this journal.

## Abbreviations

ADC: apparent diffusion coefficient
AMF: angiomyofibroblastoma
CA: cellular angiofibroma
DCE: dynamic contrast-enhanced
DW: diffusion-weighted
DWI: diffusion-weighted imaging
MR: magnetic resonance
MRI: magnetic resonance imaging
MT: magnetization transfer
MTI: magnetization transfer imaging
MTR: magnetization transfer ratio
SIm: signal intensity after the application of the saturation pulse
SIo: signal intensity without the saturation pulse
TSI: time–signal intensity

## References

[1] Nucci MR, Granter SR, Fletcher CD. Cellular angiofibroma: a benign neoplasm distinct from angiomyofibroblastoma and spindle cell lipoma. Am J Surg Pathol. 1997;21:636–44. 10.1097/00000478-199706000-00002 9199640

[2] Laskin WB, Fetsch JF, Mostofi FK. Angiomyofibroblastoma-like tumor of the male genital tract: analysis of 11 cases with comparison to female angiomyofibroblastoma and spindle cell lipoma. Am J Surg Pathol. 1998;22:6–16. 10.1097/00000478-199801000-00002 9422311

[3] Hara N, Kawaquchi M, Koike H, Nishiyama T, Takahashi K. Angiomyxoid tumor with an intermediate feature between cellular angiofibroma and angiomyofibroblastoma in the male inguinal region. Int J Urol. 2005;12:768–72. 10.1111/j.1442-2042.2005.01148.x 16174055

[4] Shintaku M, Naitou M, Nakashima Y. Angiomyofibroblastoma-like tumor (lipomatous variant) of the inguinal region of a male patient. Pathol Int. 2002;52:619–22. 10.1046/j.1440-1827.2002.01397.x 12406192

[5] Miyajima K, Hasegawa S, Oda Y, Toyoshima S, Tsuneyoshi M, Motooka M, et al. Angiomyofibroblastoma-like tumor (cellular angiofibroma) in the male inguinal region. Radiat Med. 2007;25:173–7. 10.1007/s11604-006-0114-2 17514368

[6] Maruyama M, Yoshizako T, Kitagaki H, Araki A, Igawa M. Magnetic resonance imaging features of angiomyofibroblastoma-like tumor of the scrotum with pathologic correlates. Clin Imaging. 2012;36:632–5. 10.1016/j.clinimag.2011.11.026 22920380

[7] Aytaç B, Yalçinkaya U, Vuruşkan H. Angiomyofibroblastoma-like tumor of the scrotum: a case report and review of literature. Turk Patoloji Derg. 2012;28:168–71. 10.5146/tjpath.2012.01118 22627637

[8] Dikaiakos P, Zizi-Sermpetzoglou A, Rizos S, Marinis A. Angiofibroma of the spermatic cord: a case report and a review of the literature. J Med Case Rep. 2011;5:423. 10.1186/1752-1947-5-423 PMC3179756 21878118

[9] Tsili AC, Giannakis D, Sylakos A, Ntorkou A, Sofikitis N, Argyropoulou MI. MR imaging of scrotum. Magn Reson Imaging Clin N Am. 2014;22:217–38. 10.1016/j.mric.2014.01.007 24792679

[10] Tsili AC, Argyropoulou MI, Giannakis D, Tsampalas S, Sofikitis N, Tsampoulas K. Diffusion-weighted MR imaging of normal and abnormal scrotum: preliminary results. Asian J Androl. 2012;14:649–54. 10.1038/aja.2011.172 PMC3720076 22367182

[11] Tsili AC, Argyropoulou MI, Giannakis D, Sofikitis N, Tsampoulas K. Conventional and diffusion-weighted magnetic resonance imaging findings of benign fibromatous paratesticular tumor: a case report. J Med Case Rep. 2011;5:169. 10.1186/1752-1947-5-169 PMC3113326 21539739

[12] Tsili AC, Ntorkou A, Baltogiannis D, Sylakos A, Stavrou S, Astrakas L, et al. Magnetization transfer imaging of normal and abnormal testis: preliminary results. Eur Radiol. 2015. doi:10.1007/s00330-015-3867-0. 26065397

[13] Tsili AC, Argyropoulou MI, Astrakas LG, Ntoulia EA, Giannakis D, Sofikitis N, et al. Dynamic contrast-enhanced subtraction MRI for characterizing intratesticular mass lesions. AJR Am J Roentgenol. 2013;200:578–85. 10.2214/AJR.12.9064 23436847

[14] Watanabe Y, Dohke M, Ohkubo K, Ishimori T, Amoh Y, Okumura A, et al. Scrotal disorders: evaluation of testicular enhancement patterns at dynamic contrast-enhanced subtraction MR imaging. Radiology. 2000;217:219–27. 10.1148/radiology.217.1.r00oc41219 11012448

[15] Akbar SA, Sayyed TA, Jafri SZ, Hasteh F, Neill JS. Multimodality imaging of paratesticular neoplasms and their rare mimics. Radiographics. 2003;23:1461–76. 10.1148/rg.236025174 14615558

